# Do Early Prognostic Measures Differentiate Mechanisms of ICU Death in Aneurysmal Subarachnoid Hemorrhage? An Analysis of Fatal Outcomes

**DOI:** 10.3390/jcm15197427

**Published:** 2026-09-24

**Authors:** Mustafa Ay, Serap Avcı Ay, Dilara Tüfek Öztan, Şule Asri, Ahmet Şükrü Alparslan

**Affiliations:** 1 Department of Intensive Care, Antalya Training and Research Hospital, 07100 Antalya, Türkiyedrsuleasri@gmail.com (Ş.A.); 2 Department of Anesthesiology and Reanimation, Kemer State Hospital, 07980 Antalya, Türkiye; 3 Department of Radiology, Antalya Training and Research Hospital, 07100 Antalya, Türkiye

**Keywords:** aneurysmal subarachnoid hemorrhage, intensive care unit, mortality, mechanism of death, C-reactive protein, albumin, prognostic scores

## Abstract

**Background/Objectives:** In aneurysmal subarachnoid hemorrhage (aSAH), intensive care unit (ICU) deaths may follow neurological or systemic pathways, but the relationship between early prognostic measures and the mechanism of death is unclear. **Methods:** We retrospectively reviewed 261 consecutive adults with aSAH admitted to the ICU between January 2020 and June 2026. World Federation of Neurosurgical Societies (WFNS) grade, modified Fisher scale and Subarachnoid Hemorrhage Early Brain Edema Score (SEBES) were assessed at admission; the neutrophil-to-lymphocyte ratio (NLR) and C-reactive protein (CRP)-to-albumin ratio (CAR) came from the first routine blood sample; and Acute Physiology and Chronic Health Evaluation II (APACHE II) and Sequential Organ Failure Assessment (SOFA) came from the first 24 h. Brain death was assigned to the neurological category by a predefined rule; the remaining deaths were adjudicated independently by two intensivists. **Results:** Eighty-six patients (33.0%) died: 45 (52.3%) neurological, 37 (43.0%) systemic/medical and 4 (4.7%) mixed/undetermined; 94.6% of systemic/medical deaths were sepsis-related. Agreement among the 54 independently adjudicated deaths was high (κ = 0.829). CAR discriminated toward systemic/medical death (AUC 0.671; 95% CI 0.552–0.791), whereas APACHE II (0.702) and SOFA (0.682) discriminated toward neurological death; WFNS (0.643) and SEBES (0.638) showed the same direction. NLR did not discriminate mechanism of death in this cohort (0.449). CAR was very highly correlated with CRP (ρ = 0.994). **Conclusions:** Among aSAH decedents, early prognostic measures were associated with the mechanism of death in opposite directions; the systemic/medical association predominantly reflected sepsis-related deaths. Because the analysis was restricted to decedents and time to death differed by mechanism (7 versus 26 days), the findings are exploratory and do not establish cause-specific risk prediction in the overall aSAH population.

## 1. Introduction

Aneurysmal subarachnoid hemorrhage (aSAH) remains a cerebrovascular emergency associated with high mortality despite advances in diagnosis and treatment [1]. A substantial proportion of deaths occur during intensive care unit (ICU) management. These deaths are not uniform but arise through partly distinct pathways. aSAH is considered a neurological syndrome with systemic complications; the hemorrhage triggers both neurological processes, including early brain injury, rebleeding, cerebral edema, and herniation, and systemic complications, including sepsis, respiratory failure, and organ dysfunction [2]. In cohorts in which causes of death were systematically classified, more than half of in-hospital deaths were attributed to neurological causes such as the direct effects of the primary hemorrhage and rebleeding, while a substantial proportion were attributed to medical complications [3,4]. These medical complications are common during the course of aSAH and independently worsen outcomes [5,6]. Thus, the neurological pathway arising from the brain injury itself and the systemic pathway arising from complications of critical illness represent two partly distinct sources of ICU mortality after aSAH.

Various tools are used at admission to assess prognosis and disease severity, each capturing a different aspect of the clinical process. The World Federation of Neurosurgical Societies (WFNS) grade reflects clinical neurological severity [7], the modified Fisher scale quantifies radiographic blood burden [8], and the Subarachnoid Hemorrhage Early Brain Edema Score (SEBES) reflects early cerebral edema [9,10]. These three measures primarily characterize neurological and radiological injury. In addition, general intensive care severity scores quantify multisystem disease severity and organ dysfunction [11,12,13]; however, because these scores also include a neurological component based on the Glasgow Coma Scale, they are not purely systemic measures. Routine inflammatory biomarkers such as the neutrophil-to-lymphocyte ratio (NLR) and C-reactive protein-to-albumin ratio (CAR) reflect the systemic inflammatory response, and both have been associated with adverse clinical outcomes after aSAH [14,15,16].

What these tools have in common is that they assess overall disease severity or clinical prognosis rather than the mechanism of death. However, death after aSAH does not occur through a single pathway. Therefore, an association between a given measure and poor outcome or mortality does not indicate whether that measure is related to a neurological or systemic mechanism of death. If a biomarker is elevated early, is that elevation present across all patients who subsequently die, or is it more pronounced among those who die from systemic causes? If a biomarker truly reflects the systemic pathway, its signal would be expected to be more prominent in systemic deaths. This was the question we addressed directly: among patients who died in the ICU, can systemic biomarkers distinguish systemic/medical death from neurological death, and do these biomarkers and neurological severity measures relate to the mechanism of death in the same direction, or in different directions? This distinction may be clinically relevant, as identifying early signals associated with different mechanisms of death could contribute to the future development of risk-stratification and monitoring strategies.

There is a methodological asymmetry inherent in this question, and this asymmetry shaped the study design. The association between a neurological severity score and neurological death is partly circular, because neurological death is itself defined by events related to the severity of the initial neurological insult, such as rebleeding, cerebral edema, herniation, and brain death. In contrast, the ability of systemic biomarkers to discriminate systemic death is not circular, because systemic death is defined by clinical events such as infection and organ failure rather than by the biomarker values themselves. We therefore centered the study on this non-circular relationship, namely the association between systemic biomarkers and systemic death. At the same time, systemic complications may themselves be partly related to hemorrhage severity through neurogenic and catecholamine-mediated mechanisms [17]; this connection was therefore considered in the interpretation of the findings.

The primary aim of this study was to determine, among patients with aSAH who died in the ICU, whether early prognostic measures were differentially associated with the adjudicated mechanism of death: specifically, whether systemic inflammatory biomarkers (NLR and CAR) and neurological or general ICU severity measures pointed in opposite directions for systemic/medical versus neurological death. Secondarily, we evaluated the ability of these measures to discriminate ICU mortality in the full cohort. We hypothesized that, among decedents, systemic biomarkers and neurological or general severity measures would show different directional associations with the mechanism of death. The study was designed not to develop a new prediction model or to rank competing measures, but to phenotype mortality according to its underlying mechanism.

## 2. Materials and Methods

### 2.1. Study Design and Ethics

This single-center, retrospective, observational cohort study was conducted in the Intensive Care Unit of Antalya Training and Research Hospital. Patients were admitted between 1 January 2020 and 1 June 2026, which defined the enrolment period. Follow-up was not truncated at the end of the enrolment period; each patient was followed until the ICU outcome (discharge alive or death) was determined, and all 261 patients had a documented ICU outcome. For all time-to-event descriptions, ICU admission was defined as time zero.

The study was approved by the Scientific Research Ethics Committee of Antalya Training and Research Hospital (decision no. 13/6; 7 August 2026; protocol no. 2026/325), and the requirement for informed consent was waived because of the retrospective design. The study was reported in accordance with the STROBE statement [18].

The patient population partially overlaps with that of a previously published study from our institution evaluating phase-based prediction of delayed cerebral ischemia in 216 patients with aSAH [19], which was conducted under a separate institutional ethics approval. The present study includes a broader cohort of 261 patients and addresses a distinct research question, with different primary outcomes, analytic populations, and statistical analyses. All 216 patients of that study are also included in the present cohort of 261; 45 patients are unique to the present study. The eligibility criteria also differed: the earlier study excluded patients who died or were declared brain-dead before day 5, whereas the present study applied no such restriction.

### 2.2. Patients

Consecutive adult patients (≥18 years) admitted to the ICU with a diagnosis of aSAH during the enrolment period were screened. The diagnosis of aSAH was based on demonstration of subarachnoid hemorrhage on non-contrast cranial computed tomography and confirmation of a causative ruptured aneurysm by angiographic imaging, including computed tomography angiography, digital subtraction angiography, or magnetic resonance angiography. The study included only subarachnoid hemorrhage resulting from aneurysm rupture; unruptured aneurysms and nonaneurysmal or traumatic subarachnoid hemorrhage were excluded. All 261 patients who met the eligibility criteria were included in the analysis. Patients were managed through a multidisciplinary approach involving neurosurgery, radiology, and intensive care teams in accordance with the guidelines applicable during the enrolment period; the decision to perform aneurysm treatment by surgical clipping or endovascular coiling was made on a case-by-case basis [1,20].

Complete data were available for all variables used in the primary analysis. The availability of the first routine blood sample was verified during data extraction by the presence of the required laboratory measurements in the hospital records for all 261 patients. Clinical and radiological severity measures were likewise available for all patients. Completeness of the primary analysis variables does not, however, imply completeness of all retrospective clinical information: exact blood-sampling intervals, interhospital transfer details, decisions regarding limitation of life-sustaining treatment, the specific reasons and timing of decisions not to treat the aneurysm, early seizures, and hematological comorbidity subtype were not systematically collected and could not be reliably reconstructed in all patients.

### 2.3. Variables

Neurological and radiological measures, including the WFNS grade [7], modified Fisher scale [8], and SEBES [9,10], were based on the clinical and radiological assessment at admission. In patients who underwent sedation or intubation, the WFNS grade was assigned according to the last documented neurological examination before these interventions. Modified Fisher and SEBES scores were determined from the admission computed tomography images according to their original definitions by a radiologist who was blinded to the laboratory data. Inflammatory biomarkers were derived from the first available routine blood sample obtained after hospital presentation. In patients who underwent aneurysm treatment, this sample had been obtained before surgical clipping or endovascular coiling. General ICU severity was quantified using APACHE II [11] and SOFA [12], calculated from data obtained during the first 24 h of ICU admission, using the worst values recorded within that period. Admission clinical-radiological measures, early blood biomarkers, and first-24-h ICU severity scores were collectively considered early prognostic measures in the analyses. In sedated patients, the Glasgow Coma Scale score was based on the most recent documented value before sedation rather than the observed value under sedation, thereby minimizing the influence of sedation on the neurological components of the severity scores.

Our institution serves as a referral center for aSAH, and the cohort included both patients presenting directly and patients transferred from other hospitals in the region. At our institution, blood sampling is routinely performed as part of the initial emergency evaluation of patients with suspected or confirmed aSAH. Exact sampling times, interhospital transfer status, the time of presentation to a referring hospital, pre-transfer management, and the intervals from symptom onset to hospital presentation and from hospital presentation to ICU admission were not retained as study variables; these intervals therefore could not be reconstructed reliably for the full cohort.

The primary systemic biomarkers were NLR, calculated as the absolute neutrophil count divided by the absolute lymphocyte count, and CAR, calculated as serum CRP divided by serum albumin, both derived from the same early blood sample [14,15,16]. Because albumin was measured in g/L and CRP in mg/L, CAR values in this study are approximately tenfold lower than those reported in studies expressing albumin in g/dL; this difference does not affect measures of discrimination but precludes direct comparison of cutoff values. To determine whether the signal carried by the ratio-based biomarkers originated from the ratio itself or from its individual components, CRP, albumin, lactate, and the lactate-to-albumin ratio (LAR) were examined as exploratory component analyses. Demographic characteristics, comorbidities, aneurysm and radiological features, and treatment variables were summarized descriptively but were not included in the primary models to avoid redundancy with the composite severity scores.

### 2.4. Outcomes and Classification of the Mechanism of Death

The primary outcome was the adjudicated mechanism of death among patients who died in the ICU: systemic/medical death versus neurological death. All-cause ICU mortality was considered a supportive secondary outcome to provide context for the cohort and to demonstrate the overall prognostic performance of the evaluated measures. Among decedents, the mechanism of death was classified as neurological, systemic/medical, or mixed/undetermined. Neurological death included the direct effects of the primary hemorrhage, rebleeding, new or enlarging cerebral infarction, refractory intracranial hypertension and herniation, catastrophic hydrocephalus, surgical or endovascular complications, and brain death. Systemic/medical death included sepsis or septic shock in the absence of a new catastrophic neurological event, as well as noninfectious cardiopulmonary causes—including neurogenic pulmonary edema, stress cardiomyopathy, arrhythmia, myocardial infarction, and cardiogenic shock—pulmonary embolism, and renal or hepatic failure. Sepsis and septic shock were defined according to Sepsis-3 criteria [21]. Deaths in which no dominant mechanism could be reliably determined were classified as mixed/undetermined. The complete classification criteria are provided in Supplementary Table S1; a brief decision sequence setting out the priority rules applied during adjudication is also provided there; the rubric was adapted from the pathophysiological cause-of-death categories described by Lantigua et al. [3], and the definitions of systemic death were informed by the relevant literature [17,21,22].

The classification process was conducted in two stages. First, cases with documented brain death were directly assigned to the neurological death category according to a predefined rule and were not subject to assessor interpretation, as this represented an objectively established category. Brain death was diagnosed clinically on the basis of absent brainstem reflexes together with a positive apnea test and was in all cases confirmed by cerebral angiography demonstrating absent intracranial circulation. The recorded time of death corresponded to this formal angiographic confirmation rather than to subsequent cardiac arrest.

Second, all remaining deaths were independently classified by two intensivists who were blinded to each other’s assessments. The adjudication file provided to the assessors contained the clinical course, neuroimaging findings, microbiological and infection-related records, organ-failure data, age, sex, ICU length of stay, and the admission WFNS, modified Fisher, and SEBES grades. Inflammatory biomarker values (CRP, albumin, CAR, and NLR) and the calculated APACHE II and SOFA scores were not included in the adjudication file. Assessors were instructed not to base their classification on severity grades. Interrater agreement was assessed using Cohen’s kappa based on the independent classifications obtained before consensus and was interpreted according to the Landis–Koch criteria [23]. Disagreements were resolved by consensus in a multidisciplinary panel comprising the two intensivists and one anesthesiologist. The principal investigator did not participate in the classification process.

### 2.5. Statistical Analysis

The analyses were structured hierarchically: the decedent-based mechanism analysis was designated as the primary analysis, while the remaining analyses were considered supportive or exploratory and were interpreted primarily on the basis of effect estimates and 95% confidence intervals (CIs). Continuous variables are presented as median (interquartile range, IQR) and were compared using the Mann–Whitney U test. Categorical variables were compared using the chi-square test or Fisher’s exact test, as appropriate.

In the primary decedent-based analysis, the ability of early prognostic measures to discriminate systemic/medical death from neurological death was evaluated using the area under the receiver operating characteristic curve (AUC). All AUCs were calculated with systemic/medical death defined as the positive class; therefore, values below 0.50 do not indicate absence of discrimination but rather discrimination in the direction of neurological death, and both directions are reported for clarity. Primary emphasis was placed on the direction of association and the AUC with its 95% CI. As a supporting comparison, pairwise AUC differences between CAR and WFNS, APACHE II, SOFA, and NLR were assessed using the DeLong method [24]. Because systemic/medical death was defined as the positive class, these comparisons were interpreted with respect to differences in the direction of discrimination and were not considered tests of superiority between measures. The association of neurological measures with neurological death was reported descriptively because of its partly circular nature.

Because of the limited number of events, penalized (Firth) logistic regression was used for multivariable modeling [25]. In these models, the outcome was coded as systemic/medical death (=1) and neurological death (=0). Continuous variables were standardized as z-scores within the primary decedent analysis population (*n* = 82); odds ratios therefore correspond to a one-standard-deviation increase in each predictor. CAR was entered separately into two-variable models with WFNS, APACHE II, and SOFA. Firth penalized logistic regression models were fitted using a custom implementation rather than a packaged Firth-regression function. Firth’s modified score equations were solved by Newton-Raphson iteration with step-size reduction, in which the update was halved until its largest absolute element fell below 10 (convergence tolerance, 1 × 10^−6^; maximum, 200 iterations), in Python 3.12.3 using NumPy 2.4.4 and SciPy 1.17.1. Wald-based 95% confidence intervals and two-sided *p* values were derived from the observed information matrix evaluated at the penalized maximum-likelihood estimates. The implementation and model specification are provided as Supplementary Code S1. Collinearity was assessed using the variance inflation factor (VIF). Because the distribution of CAR was markedly right-skewed, these models were repeated after natural-log transformation of CAR (transformed first, then standardized within the same population) as a sensitivity analysis. Mixed/undetermined deaths were excluded from the primary comparison and were addressed in sensitivity analyses by reclassifying them as systemic, reclassifying them as neurological, excluding rule-assigned cases, and excluding cases with assessor disagreement. Because APACHE II and SOFA include a central nervous system (CNS) component based on the Glasgow Coma Scale (GCS), their association with neurological death may be partly structural. To examine this, an exploratory analysis was performed. For SOFA, the CNS component was subtracted from the total score to derive a non-CNS SOFA score. For APACHE II, the GCS-derived neurological points (15 − GCS) were subtracted from the total score. These analyses were restricted to decedents for whom a numerical GCS value was available in the research dataset, allowing reliable reconstruction of the neurological component; the numerical pre-sedation Glasgow Coma Scale value itself was not retained as a separate research-dataset variable in all patients. Two further sensitivity analyses were performed. First, because neurological deaths occurred substantially earlier than systemic/medical deaths, a landmark analysis was restricted to decedents surviving beyond ICU day 7. Second, because patients who did not undergo aneurysm treatment were unevenly distributed between the death mechanisms, an analysis was restricted to patients who underwent surgical clipping or endovascular coiling.

The distribution of death mechanisms across admission WFNS strata was also explored. Decedents were cross-classified in a 2 × 2 table according to neurological severity at admission (low WFNS, grades 1–3, versus high WFNS, grades 4–5) and mechanism of death. The proportion of patients with low-grade neurological severity at admission who subsequently died from systemic causes was reported descriptively. This analysis was considered exploratory, and neurological severity scores were not evaluated as classifiers of the cause of death.

In the supportive analysis, AUCs for ICU mortality were calculated in the full cohort for WFNS, modified Fisher scale, SEBES, APACHE II, SOFA, NLR, and CAR. The sample size comprised all consecutive patients who met the eligibility criteria during the enrolment period. A two-sided *p* value < 0.05 was considered statistically significant. Analyses were performed using SPSS version 25 (IBM Corp., Armonk, NY, USA) and Python version 3.12.3 with NumPy version 2.4.4 and SciPy version 1.17.1.

## 3. Results

### 3.1. Cohort Characteristics and Mortality

During the enrolment period, 261 patients who met the eligibility criteria were analyzed. The median age was 57 years (IQR 47–65), and 131 patients (50.2%) were women. At admission, 100 patients (38.3%) had poor neurological grade (WFNS 4–5), and 154 (59.0%) had a high radiographic blood burden (modified Fisher grades 3–4). The aneurysm was treated by surgical clipping in 182 patients (69.7%) and by endovascular coiling in 48 (18.4%); 31 patients (11.9%) did not undergo an intervention because of their overall clinical condition. Overall, 86 patients (33.0%) died in the ICU.

For overall mortality context, compared with survivors, patients who died were older and had greater early clinical severity: median WFNS grade 4 (IQR 4–5) versus 1 (1–2), APACHE II score 20 (16–24) versus 7 (5–11), and SOFA score 5 (4–6) versus 1 (1–3) (all *p* < 0.001). Inflammatory biomarkers were also higher among decedents (NLR 14.96 vs. 9.40, *p* < 0.001; CAR 0.24 vs. 0.13, *p* = 0.003). Baseline characteristics and early measures according to survival status and mechanism of death are summarized in Table 1.

### 3.2. Classification of the Mechanism of Death and Interrater Agreement

Among the 86 deaths, brain death was documented in 32 cases (37.2%), and these cases were classified as neurological deaths according to the predefined rule. The remaining 54 deaths were independently classified by two intensivists. Raw agreement between the independent assessments was 92.6%, with a high level of interrater agreement among these 54 independently adjudicated deaths (Cohen’s κ = 0.829; 95% CI 0.668–0.990). Class-level disagreement occurred in four cases (7.4%), all of which were resolved by consensus in the multidisciplinary panel.

According to the final classification, 45 deaths (52.3%) were neurological, 37 (43.0%) were systemic/medical, and 4 (4.7%) were mixed/undetermined. Most systemic deaths were infectious in origin: 35 cases (94.6% of systemic deaths) were attributed to sepsis or septic shock; one was attributed to a noninfectious cardiopulmonary cause and one to another unclassified systemic cause. Neurological deaths comprised brain death (*n* = 32), the direct effects of the primary hemorrhage (*n* = 7), refractory cerebral edema with herniation (*n* = 4), and new or enlarging cerebral infarction (*n* = 2) (Table 2). Time from ICU admission to death differed markedly by mechanism, with a median of 7 days (IQR 3–10) for neurological deaths versus 26 days (IQR 15–44) for systemic/medical deaths (*p* < 0.001).

Among the 35 sepsis-related deaths, the documented source of infection was pulmonary in 13 (37.1%), central nervous system (ventriculitis) in 4 (11.4%), bloodstream or catheter-related in 3 (8.6%), urinary in 1 (2.9%), and multiple in 14 (40.0%). Microbiological confirmation was available in 28 patients (80.0%); among the remaining seven, four had cerebrospinal-fluid findings consistent with ventriculitis and three had radiological findings consistent with pneumonia. The first documented infection occurred at a median of ICU day 7 (IQR 6–9; range 5–18). A contributing neurological event was documented in 12 of these 35 deaths (34.3%), most commonly refractory cerebral edema or intracranial hypertension; these cases were classified as systemic/medical because the predominant mechanism proximate to death was adjudicated as systemic/medical.

Treatment and ICU-course characteristics are summarized by death mechanism in Table 3. Aneurysm treatment was not undertaken in 17 of 45 neurological deaths (37.8%) compared with 6 of 37 systemic/medical deaths (16.2%); mechanical ventilation was recorded in 38 (84.4%) and 25 (67.6%), and external ventricular drainage in 18 (40.0%) and 15 (40.5%), respectively.

### 3.3. Relationship Between Early Prognostic Measures and the Mechanism of Death

The primary analysis included 82 deaths classified as either neurological or systemic/medical (37 systemic and 45 neurological); the four mixed/undetermined cases were excluded from the main comparison. Among decedents, CAR discriminated toward systemic/medical death: median CAR was 0.39 (IQR 0.15–1.12) in systemic/medical deaths and 0.12 (0.06–0.33) in neurological deaths (*p* = 0.008), with an AUC of 0.671 (95% CI 0.552–0.791; *p* = 0.005) (Figure 1 and Figure 2). In contrast, NLR did not discriminate systemic/medical death from neurological death (AUC 0.449; 95% CI 0.321–0.576; *p* = 0.43).

Neurological and general severity measures showed discrimination in the opposite direction, indicating neurological rather than systemic death. For neurological death, the AUCs were 0.702 for APACHE II (95% CI 0.586–0.819; *p* < 0.001), 0.682 for SOFA (95% CI 0.567–0.796; *p* = 0.002), 0.643 for WFNS (95% CI 0.529–0.756; *p* = 0.014), and 0.638 for SEBES (95% CI 0.524–0.753; *p* = 0.018). The modified Fisher scale showed no discrimination in either direction (AUC 0.502; *p* = 0.98). The direction of discrimination for the evaluated measures is illustrated in Figure 3, with detailed values presented in Table 4.

The magnitude of discrimination, expressed as the absolute distance of the AUC from 0.50, was numerically comparable across measures (APACHE II, 0.202; SOFA, 0.182; CAR, 0.171; WFNS, 0.143; SEBES, 0.138); no formal equivalence testing was performed. As a supporting comparison, pairwise AUC differences between CAR and the other measures were +0.314 versus WFNS (*p* = 0.0003), +0.373 versus APACHE II (*p* < 0.001), +0.353 versus SOFA (*p* < 0.001), and +0.223 versus NLR (*p* = 0.014). These differences should not be interpreted as evidence that one measure had stronger discriminatory performance than another. For WFNS, APACHE II, and SOFA, the large pairwise differences primarily reflect the opposite directions of association relative to CAR; NLR showed little discrimination of death mechanism in this cohort.

In penalized (Firth) logistic regression models, standardized CAR and the severity measures showed associations in opposite directions when entered together. In the CAR + SOFA model, CAR was positively associated with systemic/medical death (OR 1.79; 95% CI 1.00–3.19; *p* = 0.050), while SOFA was negatively associated with systemic/medical death (OR 0.52; 95% CI 0.31–0.85; *p* = 0.010). In the CAR + APACHE II model, APACHE II retained a negative association (OR 0.50; 95% CI 0.30–0.82; *p* = 0.007), while CAR showed a borderline positive association (OR 1.70; 95% CI 0.99–2.92; *p* = 0.053). In the CAR + WFNS model, CAR again showed a borderline positive association (OR 1.64; 95% CI 0.98–2.74; *p* = 0.058), while WFNS showed a negative association (OR 0.62; 95% CI 0.39–0.98; *p* = 0.043). Variance inflation factors were below 2.6 in all models. Because the distribution of CAR was markedly right-skewed, the models were repeated after natural-log transformation of CAR. The direction of the association was preserved, with effect estimates of similar or slightly greater magnitude (ORs 1.80–1.88 per SD). All primary and log-transformed sensitivity models are presented in Table 5.

### 3.4. Mechanism of Death According to Neurological Grade at Admission

When neurological grade at admission was cross-classified with the mechanism of death, 11 of the 21 decedents with low-grade aSAH (WFNS 1–3) died from systemic/medical causes (52.4%), compared with 42.6% among the 61 decedents with high-grade aSAH (WFNS 4–5) (Fisher’s exact test, *p* = 0.458). These proportions are calculated among decedents within each grade stratum and not among all patients admitted with that grade. The distribution of death mechanisms did not differ significantly between the two strata.

Within the low-grade subgroup, CAR discriminated systemic/medical death (AUC 0.855; 95% CI 0.674–1.000; *p* < 0.001). This subgroup comprised only 21 decedents, and the analysis should be considered exploratory; it should not be interpreted as evidence that the association differs between neurological severity strata (Supplementary Table S4).

### 3.5. Component Analyses

In exploratory analyses performed to determine the source of the signal carried by the ratio-based biomarkers, CRP alone discriminated systemic/medical death to nearly the same extent as CAR (AUC 0.663; 95% CI 0.542–0.783; *p* = 0.008), with no significant difference between CAR and CRP (ΔAUC +0.008; *p* = 0.35). The correlation between CAR and CRP was very high (Spearman’s ρ = 0.994). Albumin alone (AUC 0.387; *p* = 0.074), lactate (AUC 0.403; *p* = 0.13), and LAR (AUC 0.437; *p* = 0.33) showed no discrimination. These findings indicate that the systemic-death signal captured by CAR was driven primarily by its CRP component. Additional discrimination by CAR over CRP alone was not demonstrated in this dataset, in which the two measures were very highly correlated and had closely similar AUCs; this does not establish that the ratio has no added value. Only two patients had documented infection at admission; after excluding these patients, the discriminatory ability of CAR was preserved (*n* = 80; AUC 0.657; 95% CI 0.534–0.780; *p* = 0.012). Infection at admission was identified from contemporaneous clinical and radiological documentation rather than from inflammatory biomarker levels. This analysis indicates that the finding was not driven by the two patients with documented infection at presentation, but it cannot exclude unrecognized infection, aspiration, or non-infectious inflammation related to the hemorrhage itself.

### 3.6. Sensitivity Analyses

The main finding was preserved across all sensitivity analyses (Supplementary Table S2). When cases classified according to the brain-death rule were excluded entirely and the analysis was restricted to adjudicated cases, the discriminatory ability of CAR became even more pronounced (*n* = 50; AUC 0.705; 95% CI 0.543–0.867; *p* = 0.013). Reclassification of mixed/undetermined cases in either direction and exclusion of cases with assessor disagreement likewise did not materially alter the result. Of the four cases with assessor disagreement, three were ultimately classified as mixed/undetermined and were therefore already outside the primary comparison; only one fell within the 82 patients of the primary analysis, so its exclusion left 81.

In a landmark analysis restricted to decedents surviving beyond ICU day 7 (*n* = 56; 36 systemic/medical and 20 neurological deaths), the direction of discrimination of CAR toward systemic/medical death was preserved (AUC 0.690; 95% CI 0.544–0.835; *p* = 0.011). The same was observed among decedents who underwent aneurysm treatment (*n* = 59; 31 systemic/medical and 28 neurological deaths; AUC 0.721; 95% CI 0.585–0.856; *p* = 0.001) (Supplementary Table S2). The landmark analysis does not remove the selection inherent in the decedent-based design.

Because APACHE II and SOFA include a central nervous system (CNS) component based on the Glasgow Coma Scale, their association with neurological death may be partly structural. This was examined in decedents for whom a numerical Glasgow Coma Scale value was available, which was required to reconstruct the CNS component (*n* = 52; 21 systemic/medical and 31 neurological deaths). Discrimination of SOFA toward neurological death was essentially unchanged after removal of the CNS component (AUC 0.754, 95% CI 0.619–0.890 for total SOFA versus 0.749, 95% CI 0.615–0.883 for non-CNS SOFA), whereas APACHE II showed modest attenuation (0.750, 95% CI 0.610–0.889 versus 0.699, 95% CI 0.551–0.847). Among the 30 decedents for whom the CNS component could not be reconstructed, neurological severity by WFNS was somewhat greater, whereas total APACHE II and SOFA distributions were broadly similar. These analyses remain exploratory (Supplementary Table S3).

### 3.7. Discrimination for ICU Mortality in the Full Cohort

In the full cohort, SOFA showed the highest discrimination for ICU mortality (AUC 0.893; 95% CI 0.853–0.934), followed by APACHE II and WFNS. The discriminatory performance of the inflammatory biomarkers for overall mortality was substantially lower (NLR, AUC 0.706; CAR, AUC 0.611) (Table 6).

## 4. Discussion

In this study, we directly examined the mechanisms of ICU death after aSAH and evaluated whether prognostic measures showed contrasting directional associations with the mechanism of death. ICU deaths were distributed almost evenly by mechanism, with 52.3% neurological and 43.0% systemic/medical. Against this background, the central observation was that early prognostic measures related to the mechanism of death in opposing ways: the early acute-phase response, as reflected by CAR, pointed toward systemic/medical death, while neurological and general ICU severity measures (WFNS, SEBES, APACHE II, and SOFA) pointed toward neurological death. The key point is not that one measure was superior to another, but that their associations pointed in opposite directions. A third observation qualifies the first two: the signal carried by CAR was largely driven by its CRP component, and additional discrimination by the ratio over CRP alone was not demonstrated.

Our first finding is consistent with the existing literature. In the adjudicated cohort of 1200 patients with subarachnoid hemorrhage reported by Lantigua et al., the most frequent causes of in-hospital death were the direct effects of the primary hemorrhage, rebleeding, and medical complications [3]. Causes of death and the role of withdrawal of life-sustaining treatment in the dying process have also been examined in detail in cohorts of patients with poor-grade subarachnoid hemorrhage [4]. Systemic complications are common after aSAH and have previously been shown to adversely affect outcomes [5,6,17]. The relatively high proportion of systemic deaths in our cohort (43.0%) may reflect the fact that the study population consisted exclusively of patients admitted to the ICU and therefore represented a more severely ill population; prolonged ICU stays may also increase the likelihood of developing infectious complications.

Early seizures have also been reported among the clinical features associated with very early in-hospital mortality after non-traumatic subarachnoid hemorrhage; in the series reported by Arboix and Martí-Vilalta, seizures were significantly more frequent among patients who died within the first 72 h [26]. Early seizures were not systematically collected in our dataset and could therefore not be examined in relation to the mechanism of death.

The principal contribution of this study lies in the second finding. The association of general ICU severity and organ dysfunction with mortality has been demonstrated in large cohorts [13], and CAR, NLR, CRP, and other systemic markers have also been associated with functional outcome, delayed cerebral ischemia, and overall prognosis after aSAH [14,15,16,27]. These studies established that general severity and organ dysfunction predict mortality after aSAH, and that systemic inflammatory markers relate to functional outcome and delayed cerebral ischemia. The question addressed here is different: whether such measures relate to which mechanism of death occurs among patients who ultimately die. Our data show that these measures were associated with the mechanism of death in opposite directions. This pattern persisted in the penalized regression models, in which CAR and either SOFA or APACHE II retained opposing coefficients when entered together. In terms of the magnitude of discrimination, the measures were numerically comparable; what differed was the direction of the association. At the cohort level, although higher SOFA and WFNS values were associated with all-cause mortality, among decedents higher values were associated with neurological death, whereas a greater acute-phase response at admission was associated with systemic/medical death. Whether these measures might usefully be combined is a hypothesis for future evaluation rather than a conclusion supported by the present analysis.

The two mechanisms differed markedly in their time course: neurological deaths occurred at a median of 7 days and systemic/medical deaths at 26 days. Patients dying early therefore had less opportunity to develop later systemic complications, predominantly infection, that characterized the systemic/medical group; the first documented infection occurred at a median of ICU day 7. This temporal structure reflects the clinical course but also creates inherent survival-time selection in a decedent-based comparison, because the observed associations are conditional on both death and survival long enough for a given mechanism to occur. In a landmark analysis restricted to decedents surviving beyond ICU day 7, the direction of discrimination of CAR toward systemic/medical death was preserved (AUC 0.690); however, this sensitivity analysis does not eliminate the selection inherent in the decedent-based design.

The distribution of death mechanisms across admission WFNS strata is also of clinical interest. More than half of the decedents with low-grade aSAH (WFNS 1–3) died from systemic causes. However, this proportion did not differ significantly from that observed in patients with high-grade aSAH, and the subgroup included only 21 patients. This finding should therefore be considered hypothesis-generating and requires confirmation in larger cohorts.

The inability of NLR to discriminate systemic/medical death (AUC 0.449) is a noteworthy negative finding; however, this does not imply that NLR lacks prognostic value. In aSAH, higher early NLR values have been associated with poor neurological outcomes and delayed cerebral ischemia [16]. The prognostic value of NLR has also been demonstrated in sepsis and septic shock; in a recent ICU cohort, NLR at ICU discharge was independently associated with 28-day and 6-month post-ICU mortality [28]. In our cohort, although NLR showed discrimination for all-cause ICU mortality (AUC 0.706), it did not distinguish systemic/medical from neurological death, so that mechanism-specific information could not be demonstrated for NLR in this cohort. One possible explanation is that aSAH itself induces systemic inflammation, neutrophilia, and relative lymphopenia early in its course, thereby making NLR responsive not only to systemic complications but also to the inflammatory and stress response triggered by the hemorrhage [16,17].

The interpretation of the CAR findings requires greater nuance. Previous studies, including a recent comparative biomarker study, have reported associations between CAR and adverse clinical outcomes or mortality after aSAH [14,15,29]. However, our component analyses revealed an important limitation: the signal carried by CAR was almost entirely driven by CRP (Spearman’s ρ between CAR and CRP = 0.994; ΔAUC +0.008, *p* = 0.35), whereas albumin alone showed no discrimination. This finding does not contradict previous reports supporting the prognostic value of CAR in aSAH; rather, it indicates that, in our dataset, additional discrimination by the ratio beyond CRP alone was not demonstrated. Our findings should therefore not be interpreted as evidence that CAR is a novel or superior biomarker, but rather that the acute-phase response at admission—as captured by CAR but largely driven by CRP—was associated more strongly with systemic/medical than with neurological death. A recent systematic review similarly emphasized that the evidence regarding CAR in aSAH remains limited and that prospective studies are needed [15].

The fact that 94.6% of systemic deaths were attributable to sepsis or septic shock limits the interpretation of our findings. The directional association we observed therefore applies primarily to infectious systemic death; noninfectious systemic deaths, including cardiopulmonary, thromboembolic, and organ-failure-related causes, were too infrequent in this cohort to permit separate analysis. On the other hand, this relative homogeneity may have strengthened the signal of the acute-phase marker. Neurogenic and catecholamine-mediated systemic complications are known to develop partly in relation to hemorrhage severity [17]; therefore, the systemic and neurological pathways should not be regarded as entirely independent, as they may partially overlap.

Hematological abnormalities, particularly anemia, have been associated with adverse outcome and mortality after aneurysmal subarachnoid hemorrhage [30]. In the present dataset, however, hematological disorders were not collected as a separate comorbidity category and therefore could not be examined reliably as a mortality-specific factor.

In our supportive analysis, SOFA showed the highest discrimination for all-cause mortality (AUC 0.893), consistent with previous studies emphasizing the importance of general ICU severity and organ dysfunction in aSAH [13]. In contrast, NLR and CAR showed substantially lower discrimination for overall mortality (AUCs 0.706 and 0.611, respectively). We therefore do not propose these biomarkers as new tools for overall mortality prediction; rather, their associations were more apparent in the mechanism-specific analysis than in the supportive analysis of overall mortality.

Several directions for future research emerge from these findings. First, mechanism-specific associations should be examined in the full aSAH cohort using time-to-event and competing-risk approaches that account for the different temporal profiles of neurological and systemic death. Second, prospective collection of blood-sampling times, infection onset, and treatment-limitation decisions would reduce uncertainty related to retrospective timing and selection. Third, multicenter studies including a broader range of systemic causes of death are needed to determine the generalizability of the observed pattern. Finally, whether mechanism-specific prognostic information can ultimately inform monitoring or preventive strategies remains to be established.

### Limitations

This study has several limitations. It was conducted in a single center with a retrospective design, which limits external validity and means that information not recorded in the clinical documentation could not be reflected in the analysis. The primary analysis was restricted to patients who died, and the 175 survivors were not included in the mechanism comparison. The findings should therefore not be interpreted as indicating that a patient with a high early acute-phase response can be predicted to die through a systemic pathway; they describe associations in opposite directions with the mechanism of death among patients who ultimately died, and they are conditional on both death and survival long enough for a given mechanism to occur.

Although the classification was based on a predefined rubric with independent assessment and masking of the inflammatory biomarkers and the APACHE II and SOFA scores, and showed high agreement (κ = 0.829), it inherently involved clinical judgment. Deaths with documented brain death (37.2%) were assigned by rule, which increased the size of the neurological-death group; the findings remained consistent in the sensitivity analysis excluding these cases. The adjudication file included the admission WFNS, modified Fisher, and SEBES grades as part of the clinical description, so some circularity between neurological severity and the classification of neurological death cannot be excluded. The inflammatory biomarkers and the calculated APACHE II and SOFA scores were not available to the assessors.

Several variables were not collected systematically and could not be reconstructed reliably: the exact time of blood sampling, the interval from symptom onset, interhospital transfer status and pre-transfer management, decisions to limit life-sustaining treatment, the reasons and timing of decisions not to treat the aneurysm, early seizures, and hematological comorbidity subtype. Patients who did not undergo aneurysm treatment were more frequent among neurological deaths (17 of 45) than among systemic/medical deaths (6 of 37), so treatment selection could have contributed to the observed association between neurological severity measures and neurological death. The exploratory analysis of the CNS component of SOFA and APACHE II was restricted to decedents for whom a numerical Glasgow Coma Scale value was available, which may have introduced additional selection related to data availability.

Radiological measures were assessed by a single rater, and interrater reliability was not evaluated. Because albumin was measured in g/L, CAR values cannot be compared directly with cutoff values reported in studies using other albumin units. Finally, the small number of mixed/undetermined cases (*n* = 4) precluded separate analysis of this group.

## 5. Conclusions

Among patients with aSAH who died in the ICU, early prognostic measures were associated with the mechanism of death in opposite directions. Neurological and general ICU severity measures were associated with neurological death, whereas the acute-phase response at admission—as captured by CAR and largely driven by CRP—was associated predominantly with sepsis-related systemic/medical death. Because the analysis was restricted to patients who died and the two mechanisms differed markedly in their time course, these findings should be regarded as exploratory and should not be interpreted as cause-specific risk prediction in the overall aSAH population. These results require confirmation in multicenter prospective studies that also include sufficient numbers of noninfectious systemic deaths.

## Figures and Tables

**Figure 1:**
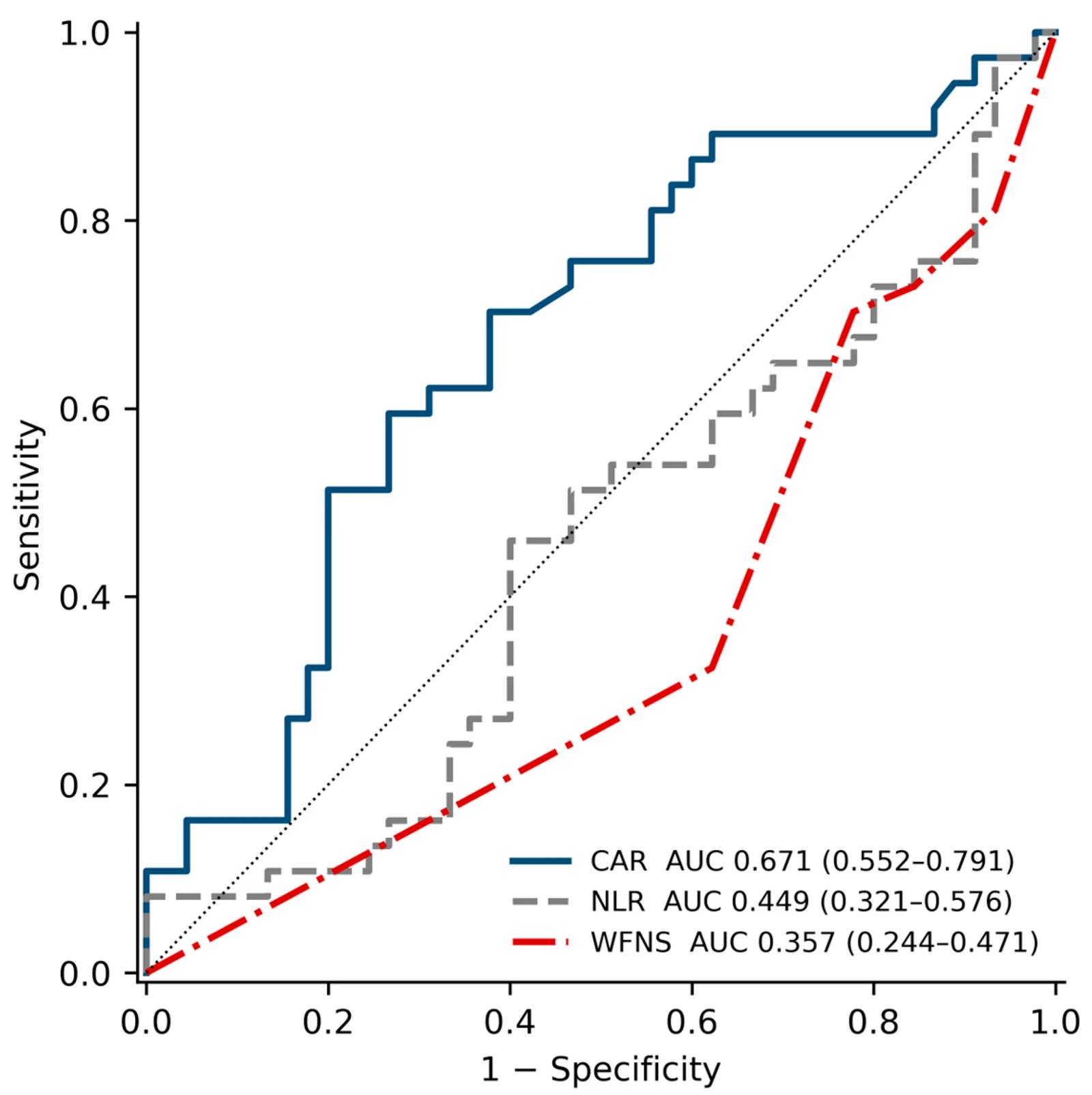
Relationship between early prognostic measures and the mechanism of death among patients who died in the ICU (ROC curves, *n* = 82). The diagonal dotted line represents no discrimination (AUC = 0.50). For all curves, systemic/medical death was defined as the positive class; values below 0.50 therefore indicate discrimination toward neurological death rather than absence of discrimination. CAR discriminated toward systemic/medical death (AUC 0.671; 95% CI 0.552–0.791). NLR discriminated in neither direction (AUC 0.449). The WFNS curve lying below the diagonal reflects discrimination toward neurological death (AUC for neurological death 0.643). CAR, C-reactive protein-to-albumin ratio; NLR, neutrophil-to-lymphocyte ratio; WFNS, World Federation of Neurosurgical Societies scale; CI, confidence interval.

**Figure 2:**
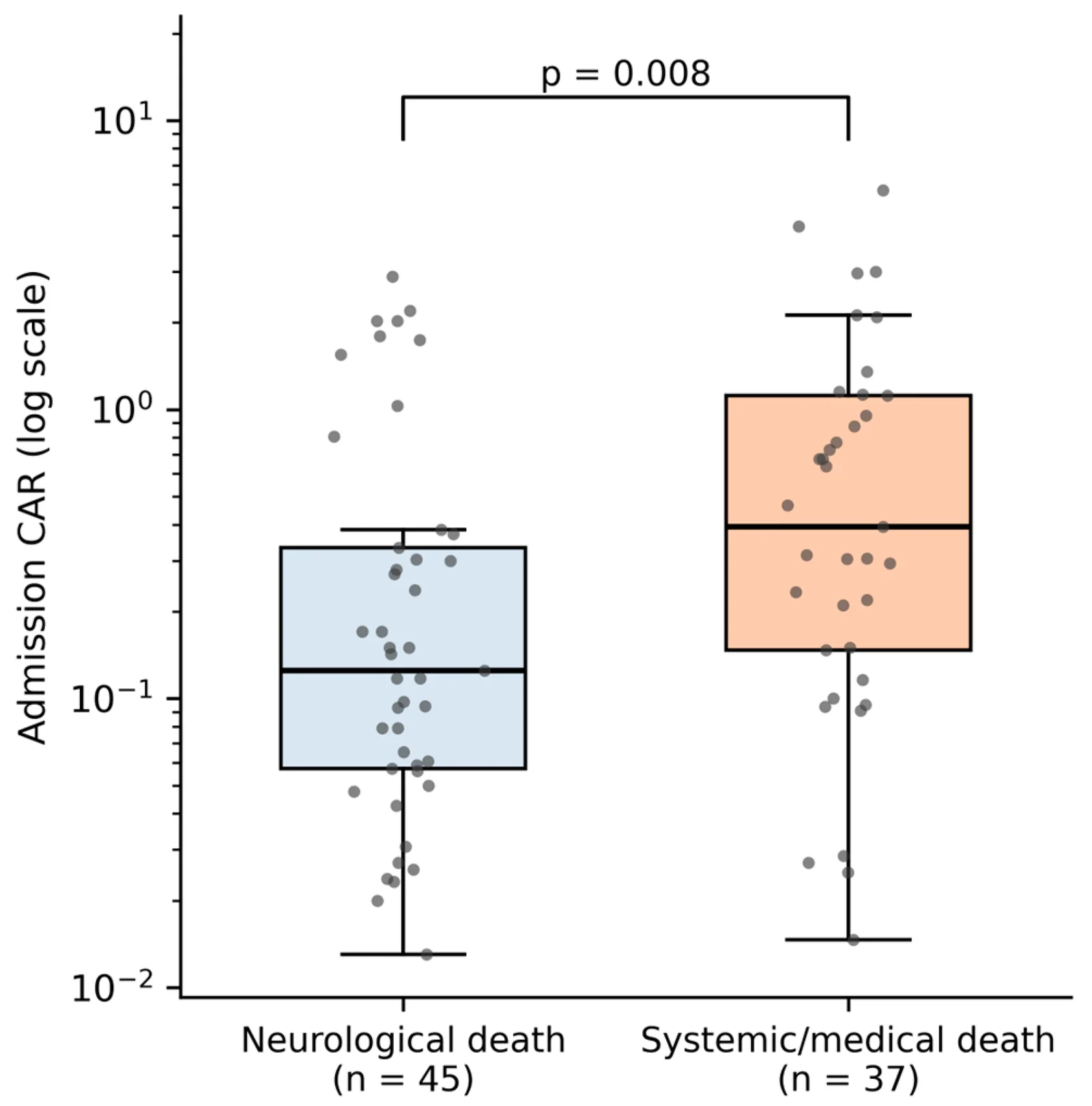
Distribution of admission CAR values according to the mechanism of death (*n* = 82). Boxes show the interquartile range, the horizontal line the median, and whiskers extend to 1.5 times the interquartile range; individual patient values are shown as dots. Median CAR was 0.39 (interquartile range 0.15–1.12) in systemic/medical deaths and 0.12 (0.06–0.33) in neurological deaths (Mann–Whitney U test, *p* = 0.008). The y axis is on a logarithmic scale.

**Figure 3:**
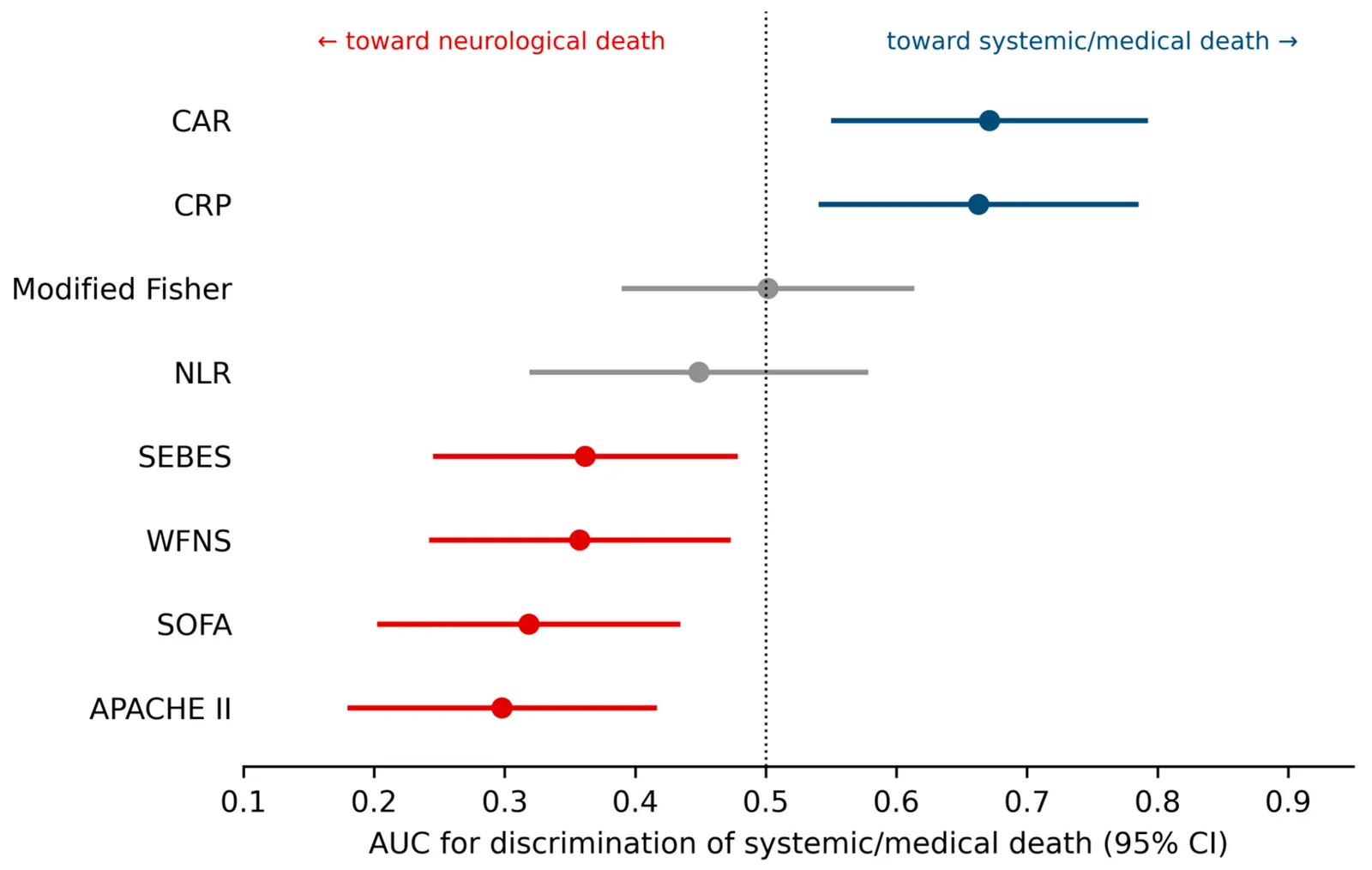
Direction and magnitude of discrimination of early measures for the mechanism of death (*n* = 82). Dots indicate the AUC for discrimination of systemic/medical death and horizontal lines the 95% confidence interval. The vertical dotted line marks the level of no discrimination (AUC = 0.50). Measures to the right of this line discriminate toward systemic/medical death (blue) and those to the left toward neurological death (red); measures whose confidence interval includes 0.50 are shown in gray. The figure illustrates the opposing directional associations of early prognostic measures with the adjudicated mechanism of death. The magnitude of discrimination (distance of the AUC from 0.50) is numerically comparable across measures; what differs is the direction of the association. No formal equivalence testing was performed.

**Table 1:** Baseline characteristics and early measures according to survival status and mechanism of death (*n* = 261).

Variable	Survivors (*n* = 175)	Neurological Death (*n* = 45)	Systemic/Medical Death (*n* = 37)
**Admission/baseline characteristics**			
Age, years	55 (47–64)	56 (47–70)	65 (57–73)
Female sex, *n* (%)	85 (48.6)	25 (55.6)	21 (56.8)
Hypertension, *n* (%)	65 (37.1)	19 (42.2)	23 (62.2)
Diabetes mellitus, *n* (%)	22 (12.6)	5 (11.1)	5 (13.5)
Coronary artery disease, *n* (%)	9 (5.1)	4 (8.9)	6 (16.2)
WFNS grade	1 (1–2)	5 (4–5)	4 (2–5)
Modified Fisher grade	2 (1–3)	4 (3–4)	4 (3–4)
SEBES	2 (1–2)	3 (3–4)	3 (2–3)
**Early biomarkers and first-24-h severity measures**			
CRP, mg/L	5 (2–11)	5 (2–13)	14 (5–38)
Albumin, g/L	39 (36–43)	39 (35–40)	35 (33–40)
CAR	0.13 (0.05–0.29)	0.12 (0.06–0.33)	0.39 (0.15–1.12)
NLR	9.40 (4.70–15.28)	14.66 (11.21–30.00)	15.37 (9.82–21.14)
APACHE II	7 (5–11)	22 (18–25)	17 (14–22)
SOFA	1 (1–3)	6 (5–7)	5 (4–5)

Data are median (interquartile range) or *n* (%). Four decedents classified as mixed/undetermined are excluded from the mechanism-specific columns. WFNS, World Federation of Neurosurgical Societies scale; SEBES, Subarachnoid Hemorrhage Early Brain Edema Score; CRP, C-reactive protein; CAR, CRP-to-albumin ratio; NLR, neutrophil-to-lymphocyte ratio; APACHE II, Acute Physiology and Chronic Health Evaluation II; SOFA, Sequential Organ Failure Assessment.

**Table 2:** Distribution of death mechanisms and predominant fatal events (*n* = 86).

Mechanism/Event	*n*	Proportion of All Deaths (%)
Neurological	45	52.3
Brain death (rule-assigned)	32	37.2
Direct effect of the primary hemorrhage	7	8.1
Refractory cerebral edema/intracranial hypertension/herniation	4	4.7
New or expanding cerebral infarction	2	2.3
Systemic/medical	37	43.0
Sepsis/septic shock	35	40.7
Cardiopulmonary (non-infectious)	1	1.2
Other systemic	1	1.2
Mixed/undetermined	4	4.7
Mixed (neurological + systemic)	2	2.3
Undetermined	2	2.3

Brain death was diagnosed and documented according to institutional criteria; these cases were assigned to the neurological category by a predefined rule and were not submitted to adjudication.

**Table 3:** Treatment and ICU-course characteristics according to the mechanism of death (*n* = 82).

Variable	Neurological Death (*n* = 45)	Systemic/Medical Death (*n* = 37)
Surgical clipping, *n* (%)	20 (44.4)	22 (59.5)
Endovascular coiling, *n* (%)	8 (17.8)	9 (24.3)
No aneurysm treatment, *n* (%)	17 (37.8)	6 (16.2)
Mechanical ventilation, *n* (%)	38 (84.4)	25 (67.6)
External ventricular drain, *n* (%)	18 (40.0)	15 (40.5)
Time from ICU admission to death, days	7 (3–10)	26 (15–44)

Data are *n* (%) or median (interquartile range). Four decedents classified as mixed/undetermined are excluded from this table. ICU, intensive care unit.

**Table 4:** Ability of early measures to discriminate the mechanism of death among decedents (*n* = 82).

Variable	Systemic/Medical Death (*n* = 37)	Neurological Death (*n* = 45)	*p* (Mann–Whitney)	AUC for Systemic/Medical Death (95% CI)	AUC for Neurological Death (95% CI)
CAR	0.39 (0.15–1.12)	0.12 (0.06–0.33)	0.008	0.671 (0.552–0.791)	0.329 (0.209–0.448)
NLR	15.37 (9.82–21.14)	14.66 (11.21–30.00)	0.428	0.449 (0.321–0.576)	0.551 (0.424–0.679)
CRP, mg/L	14 (5–38)	5 (2–13)	0.012	0.663 (0.542–0.783)	0.337 (0.217–0.458)
Albumin, g/L	35 (33–40)	39 (35–40)	0.080	0.387 (0.264–0.511)	0.613 (0.489–0.736)
WFNS grade	4 (2–5)	5 (4–5)	0.018	0.357 (0.244–0.471)	0.643 (0.529–0.756)
Modified Fisher grade	4 (3–4)	4 (3–4)	0.983	0.502 (0.391–0.612)	0.498 (0.388–0.609)
SEBES	3 (2–3)	3 (3–4)	0.024	0.362 (0.247–0.476)	0.638 (0.524–0.753)
APACHE II	17 (14–22)	22 (18–25)	0.002	0.298 (0.181–0.414)	0.702 (0.586–0.819)
SOFA	5 (4–5)	6 (5–7)	0.004	0.318 (0.204–0.433)	0.682 (0.567–0.796)

Data are median (interquartile range). AUC, area under the receiver operating characteristic curve; CI, confidence interval. Systemic/medical death was defined as the positive class; an AUC below 0.50 therefore indicates discrimination toward neurological death rather than absence of discrimination, and both directions are presented for readability. NLR and CAR were the main systemic markers; CRP and albumin were evaluated as exploratory component analyses of CAR. The *p* value for the between-group comparison was obtained using the Mann–Whitney U test and assesses the difference in distributions between systemic/medical and neurological deaths, whereas the *p* value associated with the AUC tests whether the AUC differs from 0.50.

**Table 5:** Firth penalized logistic regression models for systemic/medical versus neurological death, including log-transformed CAR sensitivity analyses.

Model	Predictor	OR (per SD)	95% CI	*p*
**Primary models**				
CAR + WFNS	CAR	1.64	0.98–2.74	0.058
	WFNS	0.62	0.39–0.98	0.043
CAR + APACHE II	CAR	1.70	0.99–2.92	0.053
	APACHE II	0.50	0.30–0.82	0.007
CAR + SOFA	CAR	1.79	1.00–3.19	0.050
	SOFA	0.52	0.31–0.85	0.010
**Sensitivity models (log-transformed CAR)**				
log(CAR) + WFNS	log(CAR)	1.80	1.11–2.89	0.016
	WFNS	0.64	0.40–1.03	0.065
log(CAR) + APACHE II	log(CAR)	1.82	1.12–2.96	0.016
	APACHE II	0.52	0.31–0.86	0.010
log(CAR) + SOFA	log(CAR)	1.88	1.15–3.08	0.011
	SOFA	0.52	0.32–0.87	0.012

All models included 82 decedents (37 systemic/medical and 45 neurological deaths). Systemic/medical death was coded as the positive outcome. Continuous predictors were standardized as z-scores within this analysis population; odds ratios therefore represent a one-standard-deviation increase. In the sensitivity models, CAR was natural-log transformed before standardization. Coefficients were estimated by Firth penalized likelihood; 95% confidence intervals and two-sided *p* values are Wald-based. CAR, C-reactive protein-to-albumin ratio; WFNS, World Federation of Neurosurgical Societies scale; APACHE II, Acute Physiology and Chronic Health Evaluation II; SOFA, Sequential Organ Failure Assessment; OR, odds ratio; SD, standard deviation; CI, confidence interval.

**Table 6:** Discrimination for all-cause ICU mortality in the whole cohort (*n* = 261, deaths = 86).

Variable	AUC (95% CI)
SOFA	0.893 (0.853–0.934)
APACHE II	0.860 (0.812–0.909)
WFNS grade	0.825 (0.772–0.879)
SEBES	0.804 (0.752–0.856)
Modified Fisher grade	0.769 (0.714–0.825)
NLR	0.706 (0.640–0.772)
CAR	0.611 (0.535–0.687)

This supportive analysis is presented for context only; the study was not designed to develop or compare prediction models.

## Data Availability

The dataset supporting the findings of this study is not publicly available because of patient confidentiality and ethical restrictions but is available from the corresponding author upon reasonable request.

## Supplementary Materials

The following supporting information can be downloaded at: https://www.mdpi.com/article/10.3390/jcm15197427/s1, Supplementary Table S1: Death mechanism classification rubric; Supplementary Table S2: Sensitivity analyses of the primary finding; Supplementary Table S3: Exploratory analysis of the central nervous system component of SOFA and APACHE II; Supplementary Table S4: Mechanism of death and admission CAR by neurological grade; Supplementary Code S1: Firth penalized logistic regression: reference implementation.
