# Supplementary material for: Do Early Prognostic Measures Differentiate Mechanisms of ICU Death in Aneurysmal Subarachnoid Hemorrhage? An Analysis of Fatal Outcomes
Source: J Clin Med. 2026 Sep 24;15(19):7427. doi: 10.3390/jcm15197427 (PMC13666138; doi:10.3390/jcm15197427)
Supplement: Supplementary file 1 [file jcm-15-07427-s001.zip › jcm-4535791-supplementary.pdf]

Supplementary Materials

Do Early Prognostic Measures Differentiate Mechanisms of ICU Death in Aneurysmal Subarachnoid Hemorrhage? An Analysis of Fatal Outcomes

Supplementary Table S1. Death mechanism classification rubric

Each death was classified according to a single predominant (proximate) mechanism. The rubric was adapted from the pathophysiological cause-of-death categories of Lantigua et al.; definitions of systemic death were based on the relevant literature.

Category	Fatal events and criteria included
Neurological	Direct effect of the primary hemorrhage; rebleeding; new or expanding cerebral infarction; refractory intracranial hypertension or herniation; catastrophic hydrocephalus; surgical or procedural complication; brain death (cases with a documented diagnosis were assigned to this category by rule).
Systemic/medical	In the absence of a proximate new catastrophic neurological event: sepsis or septic shock (irrespective of source; pulmonary, urinary, catheter-related or bloodstream); non-infectious cardiopulmonary causes (neurogenic pulmonary edema, stress cardiomyopathy, arrhythmia, myocardial infarction, cardiogenic shock); pulmonary thromboembolism; renal or hepatic failure; other systemic causes.
Mixed/undetermined	Cases in which neurological and systemic mechanisms contributed to a comparable extent (mixed), or in which the predominant mechanism could not be reliably established from the available records (undetermined).

Decision rules: (i) the predominant mechanism proximate to death was selected, and the initial precipitating event was recorded as a secondary event; (ii) for infection-related deaths meeting sepsis criteria, sepsis/septic shock was selected irrespective of source; (iii) inflammatory biomarker values (CRP, albumin, CAR, NLR) and the calculated APACHE II and SOFA scores were not included in the adjudication file, and assessors were instructed not to base their classification on severity grades; (iv) ICU length of stay alone was not used as a classification criterion.

Decision sequence: (1) Cases with formally documented brain death were assigned directly to the neurological category according to the predefined rule. (2) In the remaining cases, the predominant mechanism proximate to death was identified from the terminal clinical course. (3) If a patient had severe initial neurological injury but subsequently developed documented sepsis or septic shock that became the dominant proximate cause of terminal organ failure and death, the case was classified as systemic/medical. (4) If a recent catastrophic neurological event — rebleeding, refractory intracranial hypertension or herniation, or a new major cerebral infarction — dominated the terminal course, the case was classified as neurological. (5) If both mechanisms contributed to a comparable extent, or if no predominant proximate mechanism could be reliably established from the available records, the case was classified as mixed/undetermined. Disagreement between the two assessors was not treated as mixed; such cases were resolved by the consensus panel.

Supplementary Table S2. Sensitivity analyses of the primary finding

In all analyses the outcome was discrimination of systemic/medical death from neurological death among patients who died in the ICU.

Analysis	n (systemic/medical / neurological)	CAR AUC (95% CI)	p	ΔAUC vs WFNS	p
Main analysis (mixed/undetermined excluded)	82 (37/45)	0.671 (0.552–0.791)	0.005	+0.314	0.0003
Adjudicated cases only (rule-assigned brain death excluded)	50 (37/13)	0.705 (0.543–0.867)	0.013	+0.385	0.0002
Mixed/undetermined reclassified as systemic	86 (41/45)	0.676 (0.560–0.792)	0.003	+0.305	0.0003
Mixed/undetermined reclassified as neurological	86 (37/49)	0.655 (0.537–0.773)	0.010	+0.299	0.0005
Cases with assessor disagreement excluded	81 (36/45)	0.674 (0.554–0.795)	0.005	+0.326	0.0002
Decedents surviving beyond ICU day 7	56 (36/20)	0.690 (0.544–0.835)	0.011	—	—
Patients who underwent aneurysm treatment	59 (31/28)	0.721 (0.585–0.856)	0.001	—	—

AUC comparisons were performed using the DeLong method. ΔAUC values reflect the opposing directional associations of the measures and are not indicators of superiority. CAR, C-reactive protein-to-albumin ratio; WFNS, World Federation of Neurosurgical Societies scale; CI, confidence interval. ΔAUC comparisons were not performed for the two additional sensitivity analyses.

Supplementary Table S3. Exploratory analysis of the central nervous system component of SOFA and APACHE II

Because APACHE II and SOFA include a central nervous system (CNS) component based on the Glasgow Coma Scale (GCS), the association of these scores with neurological death may be partly structural. The analysis was restricted to decedents for whom a numerical GCS value was available in the research dataset, which was required to reconstruct the CNS component (n = 52; 21 systemic/medical and 31 neurological deaths). For SOFA, the CNS component was subtracted from the total score. For APACHE II, the GCS-derived neurological points (15 − GCS) were subtracted from the total score.

**Measure**	**AUC toward neurological death (95% CI)**	**p**
SOFA (total)	0.754 (0.619–0.890)	0.0002
SOFA (CNS component removed)	0.749 (0.615–0.883)	0.0003
APACHE II (total)	0.750 (0.610–0.889)	0.0004
APACHE II (CNS component removed)	0.699 (0.551–0.847)	0.0085

These analyses are exploratory. Within this subgroup, discrimination of SOFA toward neurological death was essentially unchanged after removal of the CNS component, whereas APACHE II showed modest attenuation. Among the 30 decedents for whom the CNS component could not be reconstructed, neurological severity by WFNS was somewhat greater, whereas total APACHE II and SOFA distributions were broadly similar.

Supplementary Table S4. Mechanism of death and admission CAR by neurological grade

Group	n	Neurological death, n	Systemic/medical death, n	Systemic/medical death, %	Median CAR, systemic/medical vs neurological death
Low grade (WFNS 1–3)	21	10	11	52.4	0.39 vs 0.06
High grade (WFNS 4–5)	61	35	26	42.6	0.48 vs 0.15

The distribution of death mechanisms did not differ significantly between groups (Fisher's exact test, p = 0.458). Within the low-grade subgroup, CAR discriminated systemic/medical death (AUC 0.855; 95% CI 0.674–1.000; p < 0.001); this subgroup comprised only 21 decedents and the analysis should be considered exploratory. It should not be interpreted as evidence that the association differs between neurological severity strata. Median admission CAR was higher in systemic/medical than in neurological deaths within both grade strata.

Supplementary Code S1. Firth penalized logistic regression: reference implementation

The Firth penalized logistic regression models reported in Table 5 were fitted using the custom Python implementation reproduced below rather than a packaged Firth-regression function. Running this code on the primary decedent analysis population reproduces all six models exactly. Environment: Python 3.12.3, NumPy 2.4.4, SciPy 1.17.1.

```
"""
Firth penalized logistic regression - reference implementation
================================================================
Supplementary code for:
  "Do Early Prognostic Measures Differentiate Mechanisms of ICU Death in
   Aneurysmal Subarachnoid Hemorrhage? An Analysis of Fatal Outcomes"

Environment : Python 3.12.3; NumPy 2.4.4; SciPy 1.17.1
Dependencies: numpy, scipy.stats (no external Firth package was used)

Estimator
---------
Firth's penalized-likelihood estimator for logistic regression, obtained by
solving the modified score equation

    U*(beta) = X' (y - p + h * (0.5 - p)) = 0,

where p = 1 / (1 + exp(-X beta)), W = diag(p(1-p)), I = X'WX, and
h = diag(W^(1/2) X I^-1 X' W^(1/2)) are the leverages. This is equivalent to
maximizing the log-likelihood penalized by Jeffreys' prior,
l*(beta) = l(beta) + 0.5 log|I(beta)|.

Solved by Newton-Raphson iteration with step-size reduction (the update is
halved until its largest absolute element falls below 10); convergence
tolerance 1e-6, maximum 200 iterations.

Inference
---------
Standard errors are taken from the inverse observed information evaluated at
the Firth estimate. Confidence intervals are Wald-type (beta +/- 1.96 SE) and
p values are two-sided Wald tests. Profile penalized-likelihood intervals were
not used.

Model specification
-------------------
Outcome : systemic/medical death = 1, neurological death = 0.
Sample  : the 82 decedents of the primary analysis (37 systemic/medical,
          45 neurological); mixed/undetermined deaths excluded.
Predictors are standardized as z-scores within this analysis population using
the population standard deviation (numpy.std, ddof=0), so that odds ratios
correspond to a one-standard-deviation increase. In the sensitivity models CAR
is natural-log transformed before standardization.
"""
import numpy as np
from scipy import stats
```

```
def firth(X, y, maxit=200, tol=1e-6):
    """Fit Firth penalized logistic regression.

    Parameters
    ----------
    X : (n, p) design matrix, first column a vector of ones (intercept)
    y : (n,) binary outcome coded 0/1

    Returns
    -------
    beta : (p,) coefficients
    se   : (p,) Wald standard errors
    pval : (p,) two-sided Wald p values
    """
    X = np.asarray(X, float)
    y = np.asarray(y, float)
    n, p = X.shape
    beta = np.zeros(p)

    for _ in range(maxit):
        eta = X @ beta
        pr = 1.0 / (1.0 + np.exp(-eta))
        W = pr * (1.0 - pr)
        XW = X * W[:, None]
        I = X.T @ XW
        try:
            Iinv = np.linalg.inv(I)
        except np.linalg.LinAlgError:
            Iinv = np.linalg.pinv(I)
        h = np.diag(XW @ Iinv @ X.T)              # leverages
        U = X.T @ (y - pr + h * (0.5 - pr))       # modified score
        step = Iinv @ U
        s = 1.0                                    # step-size reduction
        for _ in range(20):
            if np.max(np.abs(s * step)) < 10:
                break
            s /= 2.0
        beta = beta + s * step
        if np.max(np.abs(step)) < tol:
            break

    eta = X @ beta
    pr = 1.0 / (1.0 + np.exp(-eta))
    W = pr * (1.0 - pr)
    I = X.T @ (X * W[:, None])
    try:
        cov = np.linalg.inv(I)
    except np.linalg.LinAlgError:
        cov = np.linalg.pinv(I)
    se = np.sqrt(np.diag(cov))
    pval = 2.0 * (1.0 - stats.norm.cdf(np.abs(beta / se)))
    return beta, se, pval


def zscore(x):
    """Standardize within the analysis population (population SD, ddof = 0)."""
    x = np.asarray(x, float)
    return (x - x.mean()) / x.std()


def fit_model(car, covariate, y, labels):
    """Fit a two-variable model and print OR, 95% CI and p for each predictor."""
    X = np.column_stack([np.ones(len(y)), zscore(car), zscore(covariate)])
    b, se, pv = firth(X, y)
    for i, name in enumerate(labels, start=1):
        print(f"    {name:10s} OR={np.exp(b[i]):5.2f} "
              f"(95% CI {np.exp(b[i]-1.96*se[i]):.2f}-{np.exp(b[i]+1.96*se[i]):.2f}) "
              f"p={pv[i]:.3f}")


# ---------------------------------------------------------------------------
# Usage: `car`, `wfns`, `apache`, `sofa` are the admission values of the 82
# decedents; `y` is 1 for systemic/medical death and 0 for neurological death.
#
#   for name, cov in [("WFNS", wfns), ("APACHE II", apache), ("SOFA", sofa)]:
#       print(f"CAR + {name}")
#       fit_model(car, cov, y, ["CAR", name])
#
```

```
#   for name, cov in [("WFNS", wfns), ("APACHE II", apache), ("SOFA", sofa)]:
#       print(f"log(CAR) + {name}")
#       fit_model(np.log(car), cov, y, ["log(CAR)", name])
# --------------------------------------------------------------------------
```
